# Improved detection of *Escherichia coli* and coliform bacteria by multiplex PCR

**DOI:** 10.1186/s12896-015-0168-2

**Published:** 2015-06-04

**Authors:** Felipe Molina, Elena López-Acedo, Rafael Tabla, Isidro Roa, Antonia Gómez, José E Rebollo

**Affiliations:** Área de Genética, Departamento de Bioquímica y Biologia Molecular y Genética, Universidad de Extremadura, Badajoz, Spain; Dairy products, Technological institute of Food and Agriculture, Badajoz, Spain

**Keywords:** Multiplex PCR, Coliform detection, *Escherichia coli* identification

## Abstract

**Background:**

The presence of coliform bacteria is routinely assessed to establish the microbiological safety of water supplies and raw or processed foods. Coliforms are a group of lactose-fermenting *Enterobacteriaceae,* which most likely acquired the *lacZ* gene by horizontal transfer and therefore constitute a polyphyletic group. Among this group of bacteria is *Escherichia coli,* the pathogen that is most frequently associated with foodborne disease outbreaks and is often identified by β-glucuronidase enzymatic activity or by the redundant detection of *uidA* by PCR. Because a significant fraction of essential *E. coli* genes are preserved throughout the bacterial kingdom, alternative oligonucleotide primers for specific *E. coli* detection are not easily identified.

**Results:**

In this manuscript, two strategies were used to design oligonucleotide primers with differing levels of specificity for the simultaneous detection of total coliforms and *E. coli* by multiplex PCR. A consensus sequence of *lacZ* and the orphan gene *yaiO* were chosen as targets for amplification, yielding 234 bp and 115 bp PCR products, respectively.

**Conclusions:**

The assay designed in this work demonstrated superior detection ability when tested with lab collection and dairy isolated lactose-fermenting strains. While *lacZ* amplicons were found in a wide range of coliforms, *yaiO* amplification was highly specific for *E. coli*. Additionally, *yaiO* detection is non-redundant with enzymatic methods.

## Background

Public health protection requires the prompt evaluation of microorganisms in drinking water and in raw and processed foods to prevent outbreaks of microbial contamination. A broad variety of waterborne and foodborne pathogens are present at extremely low concentrations and are thus challenging to detect. Whereas coliforms [1], particularly *Escherichia coli* [2, 3], rarely cause sickness, these bacteria are abundant in human and warm-blooded animal feces and are thus regularly used as microbial indicators of the co-contaminanting presence of enteropathogenic bacteria in water and foods supplies [4].

The coliform group was vaguely defined from its inception [5], primarily by the ability of bacteria to ferment lactose, and lacks taxonomical value. Coliform bacteria are distributed among diverse genera, and different authors use different inclusion criteria [6, 7]. Nevertheless, coliforms are generally described as gram-negative, rod-shaped *Enterobacteriaceae* that ferment lactose producing acid and gas. Possession of the gene *lacZ*, which codes for the β-galactosidase, is the most prominent feature of the coliforms, whereas β-D-glucuronidase, encoded by the gene *uidA*, is routinely used to specifically identify *E. coli*. Unsurprisingly, because *uidA* and *lacZ* are paralogs [8], wild type β-D-glucuronidase also has a weak β-galactosidase activity, which is increased in some mutant alleles [9, 10]. Although lactose fermentation can be used to distinguish *Shigella* spp. from *E. coli,* several *Shigella* strains are able to ferment lactose after cultivation. This is explained by the presence of *lacZ* in specific *Shigella* genomes but the absence of LacY permease activity in culture [11].

Historically, the definition of coliforms has been primarily based on the techniques used for their detection. Traditional detection methods rely upon culturing the samples on selective media and specific incubation conditions [7, 12]. This approach allows cell enumeration but it is cumbersome, time consuming and fails to score viable but nonculturable (VBNC) bacterial cells. In *Enterobacteriaceae* gas formation from lactose is dependent on formic hydrogenlyase and easily inhibited [13]; thus, detection of coliforms by gas production lacks significance. This method also lacks specificity, as *Aeromonas* spp. can also ferment lactose [14]. Furthermore, both β-galactosidase and β-D-glucuronidase are inducible enzymes and their activity is affected by incubation temperature and the growth medium [1, 15, 16]. Consequently, both false-positive and false-negative bacteria interfere with evaluation. Several current commercial tests involve specific enzymatic assays that utilize chromogenic or fluorogenic substrates for the improved detection of coliforms [7, 17]. These methods are simple and rapid but their specificity is compromised [18, 19]. Although primarily limited to *E. coli* [20, 21], β-D-glucuronidase activity is found in other bacteria such as Flavobacteria, and it is frequent in *Yersinia, Salmonella,* and *Shigella* [3, 22]*.* Conversely, a high proportion of β-D-glucuronidase-negative *E. coli* strains has been reported [23, 24].

Exponential amplification of specific DNA sequences by PCR greatly increases the probability of detecting low concentrations of target organisms and reduces the time required to obtain results. While PCR cannot distinguish live from dead cells, it permits the detection of nonculturable cells. Moreover, PCR may yield positive results with strains that, despite bearing a gene sequence, do not express the corresponding enzyme and thus lack the corresponding enzymatic activity [24]. Multiplex PCR (mPCR) allows for the simultaneous evaluation of several strains and the detection of internal controls. Two sets of primers pairs, designed by Bej *et al.* [25, 26] for the amplification of *lacZ* and *uidA* genes, have been used to test for total coliforms and *E. coli* in clinical isolates [27] and in the upper gut contents of Lindow Man [28]. An mPCR assay was developed to target *uidA* gene for the common detection of *E. coli* and *Shigella* in milk [29]. Fricker *et al.* [30] analyzed water samples and found that only 70 % of the 324 coliform strains were correctly identified by these *lacZ* primers, whereas five non-*E. coli* coliforms were identified by *uidA* primers. These results indicate that developing alternative primer sets might be required for improved detection.

In this paper, we report a new strategy for differential bacterial identification by multiplex PCR. We wanted to combine, in a single assay, wide-range and high-specificity detection of both total coliforms and *E. coli,* respectively*.* To overcome the variability of *lacZ* sequences, primer sets were targeted to the consensus sequence of an alignment. An orphan gene, *yaiO,* was selected as the target for the specific identification of *E. coli*. The resulting amplicons, both *in silico* and *in vitro*, indicate that these probes are more efficient than those previously described.

## Results

### *yaiO* represents an alternative to *uidA* for *E. coli* identification *in silico*

The gene *yaiO* [EcoGene:EG13297]*,* selected as an alternative to *uid* amplicons, belongs to the *E. coli* orphan ORFs [31]. Nonetheless, *yaiO* transcribes in both the exponential and stationary growth phases [32], encodes a protein originally postulated by a bioinformatic prediction [33] and was later found to be expressed and localized in the outer membrane of *E. coli* [34]. These results indicate that *yaiO* corresponds to a bona fide gene and hence it might constitute an optimal target for specific *E. coli* identification by PCR detection. With this goal in mind, the oligonucleotide primer pair *yaiOF* and *yaiOR* was design to produce a 115 bp amplicon (Table 1).

**Table 1:** Oligonucleotide primers used for multiplex PCR amplification

Primer set	Source	Sequence	Product size (bp)
lacZB	Bej *et al.*, 1990	F: 5′ ATGAAAGCTGGCTACAGGAAGGCC 3′	876
		R: 5′ CACCATGCCGTGGGTTTCAATATT 3′	
lacZ3	This work	F: 5′ TTGAAAATGGTCTGCTGCTG 3′	234
		R: 5′ TATTGGCTTCATCCACCACA 3′	
uidA	Bej *et al.*, 1991	F: 5′ TGGTAATTACCGACGAAAACGGC 3′	162
		R: 5′ ACGCGTGGTTACAGTCTTGCG 3′	
yaiO	This work	F: 5′ TGATTTCCGTGCGTCTGAATG 3′	115
		R: 5′ ATGCTGCCGTAGCGTGTTTC 3′	

The Primer-BLAST tool allows to check the specificity of pre-existing primers by combining local and global alignment algorithms [35]. Therefore, we used it to compare the *in silico* PCR amplification of the *uidA* primers designed by Bej *et al.* [26] (Table 1) with the *yaiO* primer set using *Enterobacteria* as the target genomes. The resulting hits were grouped by species or genus (Table 2). Unsurprisingly, because the *Shigella* and *E. coli* lineages are very closely related [11, 36], *Shigella spp.* hits were obtained for both primer sets. However, the specificity of the *yaiO* amplification was higher 95 % of the positive hits (83 of 87) for *yaiO* primers corresponded to *E. coli*, whereas only 87.5 % of the hits (91 of 104) represented *E. coli* when the query primers were *uidA*. Ten non-*E. coli* strains rendered as hits with *uidA* primers alone, one with *yaiO,* and three were detected by both primer sets. These results suggest that although both sets are suitable for *E. coli* detection, *yaiO* might represent indeed a superior target.

**Table 2:** Comparison of expected *uidA and *yaiO PCR products by Primer-BLAST analysis

Organism	Total number of hits		*yaiO vs. uidA*		
	*yaiO +*	*uidA* +	*yaiO + uidA* -	*yaiO + uidA+*	*yaiO – uidA +*
*Escherichia coli*	83	91	31	52	39
*Escherichia* sp.	1	2	1		2
*Citrobacter rodentium*		1			1
*Shigella boydii*	1	2		1	1
*Shigella flexneri*		6			6
*Shigella sonnei*	2	2		2	
	87	104			

The number of potential target sequences yielding positive *in silico* PCR amplification (N = 136) with *yaiO* or *uidA* primers (Table 1) are grouped by species or genus. The genomes that produce single and double amplicons are compared on the right

### *lacZ* alignment and primer design

**Fig. 1:**
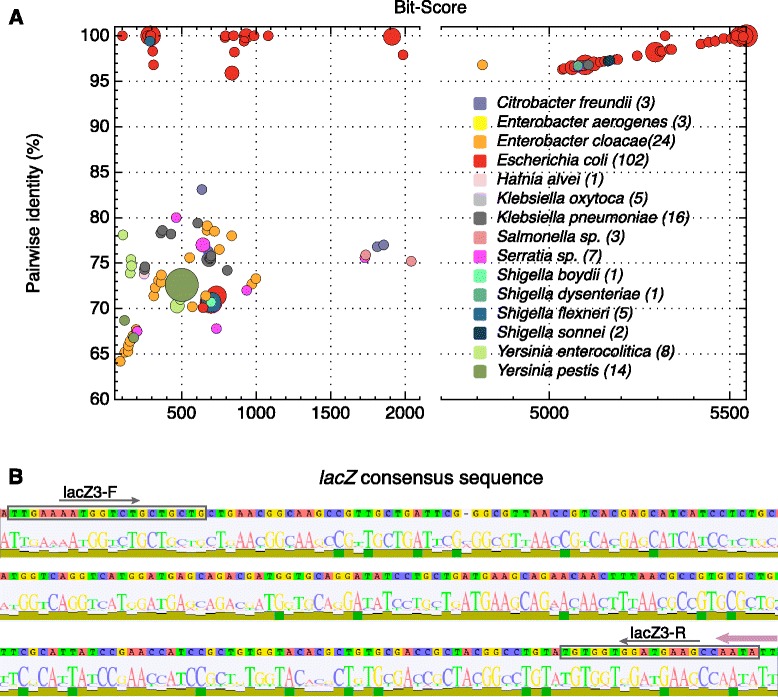
Alignment of *lacZ* sequences and designing of lacZ3 oligonucleotide primers. **a** Clusters of DNA sequences hits selected after Blast using *lacZ* from *E. coli K-12* strain MG1655 as the query sequence. 195 hits, with a minimum pairwise identity of 64 % with the query sequence, from different enterobacteria were aligned. The total number of sequences corresponding to each lineage is shown on the right in brackets. The area of each dot correlates with the number of hits from each lineage with the same identity and bit-score. **b** The consensus sequence of a *lacZ* fragment (derived from sequences outlined in panel a) was used to design LacZ3-F and LacZ3-R oligonucleotide primers. Consensus and primer sequences were obtained using ClustalW and Primer 3 software, respectively. The degree of conservation of each position in the logo sequence is shown by the relative height of each base. The lacZ3R primer binding site overlaps with 3’ end of the lacZB (Bej *et al.*, [25, 26]) primer (indicated by a pink arrow)

By performing a comparative analysis of an assortment of *lacZ* sequences and developing new PCR primers, it may be possible to improve the accuracy of coliform detection. The DNA sequence of the *lacZ gene* from the *E. coli* strain MG1655 [EcoGene:EG10527] was used to perform a BLASTn search. From the resulting hits, a total of 195 sequences (with a minimum identity of 64 %) were selected, and a *lacZ* consensus sequence was obtained running ClustalW [37] software. Considering the ambiguous definition of the coliform group, we did not restrict the sequences to historical coliforms but incorporated sequences from other enterobacteria (Fig. 1a). The statistical significance of the alignments was evaluated by comparing the pairwise identity (%) and and the bit-scores of the selected BLAST hits, revealing three clusters of sequences. Although most *E. coli* sequences showed high identity (above 95 %), some possessed high bit-scores (above 5000), showing a linear correlation between identity and bit-score (Fig. 1a, top right), whereas others depict lower values (below 2000). These groups correspond to “full” and “incomplete” β-galactosidase genes, respectively. Strikingly, the bulk of the sequences from other lineages forms a third group with lower identity (below 85 %) and bit-score values. Although the lack of lactose fermentation is commonly used to distinguish *Shigellae* from *E. coli* [11, 38], some *Shigella* strains harbor the gene *lacZ* (see Background). In the analysis, BLAST hits were obtained for several *Shigella* lineages, but the hits found for *Shigella dysenteriae* and *Shigella sonnei* coincide with the gene *ebgA,* which is paralogous with *lacZ*. These results indicate that designing PCR primers by using a *lacZ* consensus sequence as a target might widen the spectrum of coliform detection. Because similar sized amplicons are expected to work better on multiplex PCR [39], we designed the primer set to produce an amplicon that was similar in size to but distinguishable from the *yaiO* amplicon (Table 1). Therefore, lacZ3 oligonucleotide primers (Table 1) were designed to amplify a highly conserved zone of *lacZ*, resulting in a 234 bp PCR product (Fig. 1b).

### Specificity of *E. coli* and coliform detection using yaiO, uidA and lacZ primer sets for multiplex PCR

**Fig. 2:**
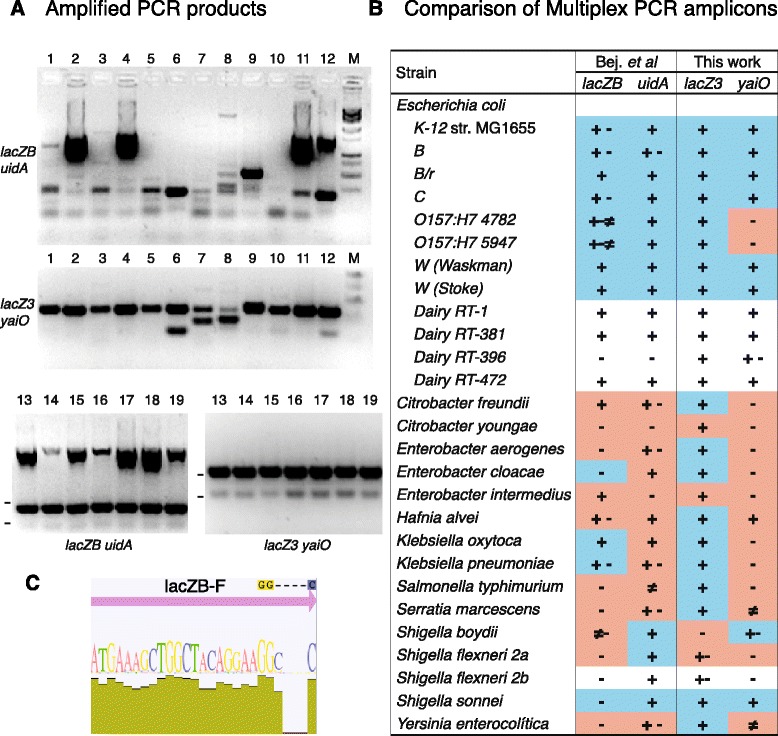
Comparison of lacZ3-yaiO and lacZB-uidA primer sets for *E. coli* and coliform identification. **a** Agarose gels (1.5 %) electrophoresis showing representative multiplex PCR amplified products from bacterial DNA. Lanes: 1, *Klebsiella pneumoniae;* 2, *Klebsiella oxytoca;* 3,*Enterobacter aerogenes*; 4, *Enterobacter intermedius;* 5, *Enterobacter cloacae;* 6, *Shigella sonnei;* 7, *Serratia marcenses;* 8, *Yersinia enterocolitica;* 9*, Salmonella typhymurium;* 10, *Citrobacter youngae;* 11; *Citrobacter freundii;* 12, *Hafnia alvei;* M, molecular weight marker (1Kb Plus DNA ladder); 13, *Escherichia coli K-12;* 14, *E. coli B;* 15, *E. coli B/r;* 16, *E. coli C;* 17, *E. coli W (Waskman);* 18, *E. coli W (Stoke);* 19, *E. coli RT1.* The oligonucleotide primer pairs used are indicated on the left or below each picture. For size comparison, the locations of 100 and 200 bp bands are shown when the marker is omitted. **b** *In vitro* and *in silico* comparison of lacZ3-yaiO and lacZB-uidA multiplex PCR amplicons. *In silico* analysis (see Methods) is indicated by color shading. Cyan: positive amplification. Light red: no amplification. White: Not tested. Each PCR reaction was carried out four times. *In vitro* PCR products are shown by signs indicating the percentage of positive amplifications obtained. “**+**”; “**+ −** “; and “**-**“ represent 100 %, 50 % and 0 % positive results, respectively. No other values, (*i.e.*, neither 75 % nor 25 % positive amplifications) were obtained. “**≠**“ indicates a different sized PCR product. **c** The 3’ end of the lacZB-R primer binds to a zone of low conservation. From top to bottom (arbitrary scale), each panel depicts the binding sites of the lacZ primers, the consensus sequence of *lacZ*, its coverage considering all the sequences aligned, sequence logo, and % identity

The performance of two pairs of oligonucleotide primers, named here lacZB-uidA, developed by Bej *et al.* [25, 26] for coliform detection by multiplex PCR, was compared with the newly designed lacZ3-yaiO primers (Table 3). To this end, *in vitro* multiplex PCR amplification and *in silico* PCR simulation were conducted (Materials & Methods). *In silico* evaluation is utilized to elucidate the source of false positive and false negative results obtained with *in vitro* experiments [40]*.* PCR reactions were carried out with culture collection and dairy isolated bacterial strains (Table 3) that had undergone total DNA extraction. Optimal thermocycling conditions with lacZ3-yaiO primers were determined by varying both the annealing temperature and extension time until best results were obtained (materials & methods). Amplified DNA was evaluated for the expected products using agarose electrophoresis (Fig. 2a) and compared with *in silico* simulations (Fig. 2b). Simultaneous amplifications with lacZB-uidA were also performed following the method described by Tantawiwat *et al.* [27]. Although described as an optimized protocol, we did not note any improvements using such thermocycling setup (data not shown).

**Table 3:** Bacterial strains used in this work

Organism	Source^a^
*Escherichia coli* K12 (MG1655)	This Lab (Molina *et al.* [52])
*Escherichia coli* B (Luria)	CECT4201
*Escherichia coli* B/r	CECT105
*Escherichia coli* C (Sinsheimer)	CECT4622
*Escherichia coli* O157:H7	CECT4782
*Escherichia coli* O157:H7	CECT5947
*Escherichia coli* W (Waskman)	CECT99
*Escherichia coli* W (Stoke)	CECT727
*Escherichia coli* RT1	Torta del Casar cheese
*Escherichia coli* RT381	Torta del Casar cheese
*Escherichia coli* RT396	Torta del Casar cheese
*Escherichia coli* RT472	Torta del Casar cheese
*Citrobacter freundii*	CECT7464
*Citrobacter youngae*	CECT5335
*Enterobacter aerogenes*	CECT684
*Enterobacter cloacae* (RT102)	Ibores cheese
*Enterobacter intermedious*(RT38)	Ibores cheese
*Hafnia alvei*	CECT 158
*Klebsiella oxytoca* (RT30)	Ibores cheese
*Klebsiella pneumoniae* ssp. *pneumoniae*	CECT143
*Salmonella typhimurium*	CECT722
*Serratia marcescens* spp. *marcescens*	CECT846
*Shigella boydii*	CECT583
*Shigella flexneri 2a*	CECT585
*Shigella flexneri 2b*	CECT4804
*Shigella sonnei*	CECT4887
*Yersinia enterocolitica ssp.enterocolitica*	CECT4315

^a^CECT = Colección Española de Cultivos Tipo, Burjasot, Valencia, Spain

Torta del Casar is made from raw milk of sheep from the Merino and Entrefino breeds

Ibores cheese is made from whole, raw milk from goats of the Serrana, Verata and Retinta breeds and their crossbreeds

Overall, the lacZ3 primers showed superior identification efficiency for *E. coli* and coliform bacteria. In agreement with the *in silico* results (Fig. 2), most *E. coli* samples generated the expected PCR product with any *lacZ* primer set, although the lacZB band was very weak or repeatability was compromised (50 % or less amplification success) in several strains, whereas the lacZ3 primers robustly identified all of the *E. coli* strains evaluated. Interestingly, additional nonspecific bands were exclusively detected when amplification was performed with lacZB-uidA and the target was not *E. coli.* Furthermore, although lacZB did not produce the expected amplicon for several coliform lineages (*Citrobacter youngae, Enterobacter aerogenes, Enterobacter cloacae, Salmonella typhymurium, Shigella spp.* and *Yersinia enterocolitica)*, amplification with lacZ3 resulted in the expected 234 bp band with all samples except *Shigella boydii* and *Sh. flexneri*.

## Discussion

Accurate molecular detection of *E. coli* is critical for the food industry because this bacterium is considered a primary faecal indicator. However, though it is widely assayed, neither β-D-glucuronidase activity nor *uidA* amplification unequivocally identify *E. coli* (see Background). Therefore, we wanted to design a PCR primer set non-redundant with the target of the enzymatic tests, *i.e.*, an alternative to *uid* amplicons. To this end, we chose the gene *yaiO,* which exhibits no significant similarity to any other real or hypothetical gene [31]. Although orphan genes show a narrow distribution among *E. coli* genomes, with most of them being found in only a single genome [41], *yaiO* shows a wide within-species distribution. Essential orphan genes would be ideal targets for the highly specific identification of all *E. coli* serotypes, but comparative genomics has revealed a clear conservation tendency of essential *E. coli* genes throughout the bacterial kingdom [31]. Consequently, neither *uidA* nor *yaiO* are essential. Nonetheless, the specificity of *E. coli* identification was higher with *yaiO* primers (Fig. 2b). In agreement with our results, an evaluation of putative orthologs of *E. coli* genes revealed that *yaiO* depicts a lower evolutionary retention index than does *uidA* [31], suggesting lower conservation and higher specificity of *yaiO.* Although Bej *et al.* [26] reported higher specificity with *uidA* primers than determined in our study*,* Fricker *et al.* [30] found false-positive *uidA* amplification using both *H. alvei and Serratia odorifera.* Several coliform bacteria (*Citrobacter freundii*, *Ent. aerogenes, Ent. cloacae, Klebsiella pneumoniae* and *Sh. flexneri)* produced the amplicon expected for *E. coli*, (*i.e.* false positives) exclusively with *uidA* primers, whereas others yielded amplicons of unexpected sizes with *uidA* (*S. thypimurium)* or *yaiO* (*Serratia marcescens* and *Yersinia pestis*). Finally, DNA from *H. alvei, Sh. boydii* or *Sh. sonnei* generated amplicons with both yaiO and uidA primers. Though it is considered an orphan gene, *yaiO* appears in some *Shigella* strains. However, *uidA* is present in the three *Shigella* species analyzed (Table 2 and Fig. 2b). This is not surprising because genetic variation within the four species of *Shigella* is encompassed within the range found in natural populations of *E. coli*. In fact, the *Shigella* genus has been proposed to have evolved from multiple *E. coli* strains after the divergence of the O157 and K12 lineages [42]. Our results support that the *Shigellae* should more aptly be classified as pathogenic sublineages of *E. coli* that acquired virulence factors by lateral gene transfer [43]*.*

*E. coli* serogroup O157:H7 is the pathogen that is most commonly associated with foodborne disease outbreaks. However, O104:H4 is an emerging strain that was identified in the 2011 German epidemic [44] and could become more prevalent in the future. A +93 *uidA* single nucleotide polymorphism has been used to characterize [45, 46] and differentiate the O157 serogroup from O104:H4 [44]. Additionally, two frameshift mutations in the *uidA* structural gene account for the absence of glucuronidase activity in O157:H7 isolates [47]. Interestingly, these strains could also be differentiated by yaiO amplification. In agreement with the Primer-BLAST results and the absence of *yaiO* (data not shown), the O157:H7 strains did not render any PCR product with *yaiO* primers (Fig. 2b). Conversely, the O104:H4 strains harbor *yaiO* and gave positive amplification on the Primer-BLAST analysis with *yaiO* primers.

Detecting coliforms for food safety or epidemiological purposes requires an understanding of the manner in which genes are acquired and evolved because these processes may allow for the colonization of new niches and adaptation to their hosts and may possibly lead to speciation events. The lactose operon was likely acquired via horizontal transfer by unrelated bacterial lineages [48], thus hindering the establishment of alternative targets for coliform detection. Some lactose-negative strains such as *Sh. boydii* and *Sh. flexneri* lack *lacZ* but incorporate the paralogous gene *ebgA* [11]*,* which explains the difference between the positive BLAST hits (Fig. 1a) and the negative or inconsistent amplification (Fig. 2b). Conversely, *Sh. sonnei*, which is considered lactose negative due to inactivation of the permease LacY [49, 50], harbors the *lacZ* gene, as confirmed by amplification with lacZ3 (Fig. 2b). Similarly, *S. typhimurium* and *Hafnia alvei,* in which *lacZ* has become a pseudogene, were identified by lacZ3*.* Likewise, some lactose-positive *Salmonella* strains, despite being considered non-coliforms, have been found [51]*.* All behavioral differences between the lacZ3 and lacZB primers pairs could be ascribed to the variability of the *lacZ* sequence, notably at the 3’ end of the forward lacZB primer (relevant for the extension of the PCR), which binds to a site of low conservation in the consensus sequence of *lacZ* (Fig. 2c). On the contrary, the binding sites of both lacZ3 primers are highly conserved (Fig. 1b). Additionally, the homology between *lacZ* and *uidA* sequences and/or the size of the amplicons [39] might contribute to diminish the performance of lacZB-uidA*.*

## Conclusion

Horizontal gene transfer entails the high evolvability of bacterial genomes but hampers the specific detection of indicators such as coliforms. However, when the food industry involves complex bacterial activity, such as cheese production, the accuracy of the bacterial detection is crucial. Amplification of DNA sequences by PCR allows the detection of nonculturable or dead cells. The primers designed in this work, which target a conserved region of *lacZ* and the orphan gene *yaiO,* demonstrated superior detection ability when tested with laboratory collection and lactose-fermenting strains isolated from dairy samples.

## Methods

### Bacterial strains, growth conditions and DNA extraction

A total of 24 bacterial strains were used in this work (Table 3). Most reference strains were obtained from the CECT (Colección Española de Cultivos Tipo, Valencia, Spain). The *E. coli K-12* strain belongs to our lab collection [52] (Molina *et al.*, [52]). The rest of the strains were isolated from raw milk cheese corresponding to Torta del Casar and Queso Ibores Protected Designation of Origins. Samples were taken from milk, curd and cheese at different ripening times, and the isolates were identified (data not shown) using the EnteroPluri-Test system (Liofilchem, Roseto degli Abruzzi, Teramo, Italy), the Biolog Microbial ID system (Biolog, Inc., Hayward, CA, USA), and one dimensional sodium dodecyl sulphate-polyacrylamyde gel electrophoresis (SDS-PAGE) of whole-cell protein [53]. All *E. coli* strains were grown at 37 °C in Lysogeny Broth medium medium, whereas other strains were cultured as described elsewhere (http://www.straininfo.net and http://www.cect.org/bacterias.php). Isolation of DNA from bacterial cells was performed using STE buffer (100 mM NaCl, 10 mM Tris buffer, pH 7.0, 1 mM EDTA) as described elsewhere [54], followed by ethanol precipitation. The concentration and quality of the DNA were determined by a spectrophotometer NanoDrop. 2000c (Thermo Fisher Scientific, Waltham, MA, USA). The DNA preparations were stored at −20 °C until use.

### Primer development

All of the oligonucleotides primers used in this study were synthesized by IDT (Integrated DNA Technologies, San Diego, CA, USA) and are listed in Table 1. Newly designed primer sequences were obtained using the Primer3 web interface [55, 56]. The DNA sequences of *yaiO* and *lacZ* genes from *E. coli* strain MG1655 were used to design *yaiO* primer pairs and as queries to perform a BLASTn analysis [57] respectively. Of the BLAST hits obtained, 195 sequences corresponding to enterobacteria were selected, and a *lacZ* consensus sequence was then determined using ClustalW [37]. The conserved regions of *lacZ* were used as template to design lacZ3 primers.

### *In silico* specificity test

The putative amplicons that could be generated by the *yaiO* and *uidA* primers were evaluated using the Primer-BLAST tool [35], restricting the target templates to *Enterobacteriaceae*. Low primer specificity stringency was set, and only targets with nine or more mismatches were ignored.

When available, full genome sequences of the bacterial strains shown in Table 3 were downloaded from the NCBI servers. Primer3 and MPrimer [58] were used to evaluate the *in silico* amplification with the oligonucleotide primers shown in Table 1.

### PCR optimization and conditions

All PCRs were adjusted to 50 μl with RNase-free water and contained 1 μl of 10 mM dNTP mix, 125 nM of each required oligonucleotide primer, 1.25 U of DNA polymerase (iTaq, Bio-Rad), 30 ng of template DNA and 1X PCR reaction buffer (20 mM Tris–HCl pH 8.4, 50 mM KCl). To optimize the multiplex amplification, the concentration of MgCl_2_ and the annealing and extension temperatures were varied (data not shown). The best results were achieved under the following conditions: 1.5 mM MgCl_2_, initial denaturation at 95 °C for 3 min, followed by 35 cycles of denaturation at 95 °C for 30 s, primer annealing at 58 °C for 30 s, primer extension at 72 °C for 1 min, and a final extension at 72 °C for 10 min. In every assay, a buffer control, to which no DNA template was added, was used as a negative control. To evaluate its reproducibility, all multiplex PCRs were performed four times, twice on an iCycler iQ system (Bio-Rad, Hercules, CA, USA) and twice with a Veriti-96 Well Thermal Cycler (Applied Biosystems, Carlsbad, CA, USA). Additionally, amplifications with lacZB-uidA were carried out as described elsewhere [27]. Briefly, the thermocycling conditions were as follows: initial denaturation at 94 °C for 10 min, followed by 44 cycles of denaturation at 94 °C for 1 min, primer annealing at various temperatures (2 cycles at 62 °C, 2 cycles at 61 °C, 2 cycles at 60 °C, 2 cycles at 59 °C and 36 cycles at 58 °C) for 1 min, primer extension at 72 °C for 1 min, and a final extension at 72 °C for 10 min.

### Detection of amplified DNA

Twenty μl of the PCR amplified product were separated by electrophoresis on 1.5 % agarose gel in 1X TAE buffer (40 mM Tris-base, 20 mM acetic acid, and 1 mM EDTA pH 8.0). The gel was stained with ethidium bromide (0.5 μg/ml) or 1X SYBR Green (Life Technologies), analyzed using a GelDoc XR (Bio-Rad, Hercules, CA, USA) transilluminator and photographed with a digital camera using Quantity One 4.6.9. The 1Kb Plus DNA ladder (Life Technologies Co., Carlsbad, CA, USA) was used as a molecular marker to indicate the size of the amplicons.

## References

[1] Leclerc H, Mossel DAA, Edberg SC, Struijk CB (2001). Advances in the bacteriology of the coliform group: their suitability as markers of microbial water safety. Annu Rev Microbiol.

[2] Bredie WL, de Boer E (1992). Evaluation of the MPN, Anderson-Baird-Parker, Petrifilm *E. coli* and Fluorocult ECD method for enumeration of *Escherichia coli* in foods of animal origin. Int J Food Microbiol.

[3] Feng PC, Hartman PA (1982). Fluorogenic assays for immediate confirmation of *Escherichia coli*. Appl Environ Microbiol.

[4] McFeters GA, Bissonnette GK, Jezeski JJ, Thomson CA, Stuart D (1974). Comparative survival of indicator bacteria and enteric pathogens in well water. Appl Microbiol.

[5] Parr LW (1939). Coliform bacteria. Bacteriol Rev.

[6] Rice, EW, Baird, RB, Eaton, AD, Clesceri, LS, editors. (2012) Standard Methods for the Examination of Water & Waste- water. 22nd edition. Washington, American Public Health Association (APHA), American Water Works Association (AWWA) & Water Environment Federation (WEF).

[7] Rompré A, Servais P, Baudart J, de-Roubin MR, Laurent P (2002). Detection and enumeration of coliforms in drinking water: current methods and emerging approaches. J Microbiol Methods.

[8] Henrissat B (1991). A classification of glycosyl hydrolases based on amino acid sequence similarities. Biochem J.

[9] Geddie MLM, Matsumura II (2004). Rapid evolution of beta-glucuronidase specificity by saturation mutagenesis of an active site loop. J Biol Chem.

[10] Matsumura I, Ellington AD (2001). *In vitro* evolution of beta-glucuronidase into a beta-galactosidase proceeds through non-specific intermediates. J Mol Biol.

[11] Yang F, Yang J, Zhang X, Chen L, Jiang Y, Yan Y (2004). Genome dynamics and diversity of *Shigella* species, the etiologic agents of bacillary dysentery. Nucleic Acids Res.

[12] Edberg SC, Allen MJ, Smith DB (1988). National field evaluation of a defined substrate method for the simultaneous enumeration of total coliforms and *Escherichia coli* from drinking water: comparison with the standard multiple tube fermentation method. Appl Environ Microbiol.

[13] Gleeson C, Gray NF (1997). The coliform Index and waterborne disease : problems of microbial drinking water assessment.

[14] Hazen TC, Fliermans CB, Hirsch RP, Esch GW (1978). Prevalence and distribution of *Aeromonas hydrophila* in the United States. Appl Environ Microbiol.

[15] Leclerc H, Mossel DA, Trinel PA, Gavini F. Microbiological monitoring- a new test for fecal contamination, Bacterial indicators, health hazards associated with water. A.W. Hoodley and B.J. Dutka eds. 1977. p. 23–36.

[16] Tryland I, Fiksdal L (1998). Enzyme characteristics of β-D-galactosidase-and β-D-glucuronidase-gositive gacteria and their interference in rapid methods for detection of waterborne coliforms and *Escherichia coli*. Appl Environ Microbiol.

[17] Muirhead RW, Littlejohn RP, Bremer PJ (2004). Evaluation of the effectiveness of a commercially available defined substrate medium and enumeration system for measuring *Escherichia coli* numbers in faeces and soil samples. Lett Appl Microbiol.

[18] Fiksdal L, Tryland I (2008). Application of rapid enzyme assay techniques for monitoring of microbial water quality. Current Opin Biotech.

[19] Poucke SOV, Nelis HJ (1997). Limitations of highly sensitive enzymatic presence-absence tests for detection of waterborne coliforms and *Escherichia coli*. Appl Environ Microbiol.

[20] Hartman PA (1989). The MUG (glucuronidase) test for *Escherichia coli* in food and water.

[21] Kaspar CW, Hartman PA, Benson AK (1987). Coagglutination and enzyme capture tests for detection of *Escherichia coli* beta-galactosidase, beta-glucuronidase, and glutamate decarboxylase. Appl Environ Microbiol.

[22] Frampton EW, Restaino L (1993). Methods for *Escherichia coli* identification in food, water and clinical samples based on beta-glucuronidase detection. J Appl Microbiol.

[23] Chang GW, Brill J, Lum R (1989). Proportion of beta-D-glucuronidase-negative *Escherichia coli* in human fecal samples. Appl Environ Microbiol.

[24] Feng P, Lum R, Chang GW (1991). Identification of *uidA* gene sequences in beta-D-glucuronidase-negative *Escherichia coli*. Appl Environ Microbiol.

[25] Bej AK, Steffan RJ, DiCesare J, Haff L, Atlas RM (1990). Detection of coliform bacteria in water by polymerase chain reaction and gene probes. Appl Environ Microbiol.

[26] Bej AK, DiCesare JL, Haff L, Atlas RM (1991). Detection of *Escherichia coli* and Shigella spp. in water by using the polymerase chain reaction and gene probes for uid. Appl Environ Microbiol.

[27] Tantawiwat S, Tansuphasiri U, Wongwit W, Wongchotigul V, Kitayaporn D (2005). Development of multiplex PCR for the detection of total coliform bacteria for *Escherichia coli* and *Clostridium perfringens* in drinking water. Southeast Asian J Trop Med Public Health.

[28] Fricker EJ, Spigelman M, Fricker CR (1997). The detection of *Escherichia coli* DNA in the ancient remains of Lindow Man using the polymerase chain reaction. Lett Appl Microbiol.

[29] Riyaz-Ul-Hassan S, Syed S, Johri S, Verma V, Qazi GN (2009). Application of a multiplex PCR assay for the detection of *Shigella*, *Escherichia coli* and Shiga toxin-producing *Esch. coli* in milk. J Dairy Res.

[30] Fricker EJ, Fricker CR (1994). Application of the polymerase chain reaction to the identification of *Escherichia coli* and coliforms in water. Lett Appl Microbiol.

[31] Gerdes SY, Scholle MD, Campbell JW, Balazsi G, Ravasz E, Daugherty MD (2003). Experimental determination and system level analysis of essential genes in *Escherichia coli* MG1655. J Bacteriol.

[32] Alimi JP (2000). Reverse transcriptase-polymerase chain reaction validation of 25 “orphan” genes from *Escherichia coli* K-12 MG1655. Genome Res.

[33] Casadio RR, Fariselli PP, Finocchiaro GG, Martelli PLP (2003). Fishing new proteins in the twilight zone of genomes: the test case of outer membrane proteins in *Escherichia coli* K12, *Escherichia coli* O157:H7, and other Gram-negative bacteria. Protein Sci.

[34] Marani P (2006). New *Escherichia coli* outer membrane proteins identified through prediction and experimental verification. Protein Sci.

[35] Ye J, Coulouris G, Zaretskaya I, Cutcutache I, Rozen S, Madden TL (2012). Primer-BLAST: a tool to design target-specific primers for polymerase chain reaction. BMC Bioinformatics.

[36] Jin Q, Yuan Z, Xu J, Wang Y, Shen Y, Lu W (2002). Genome sequence of *Shigella flexneri* 2a: insights into pathogenicity through comparison with genomes of *Escherichia coli* K12 and O157. Nucleic Acids Res.

[37] Ramu C, Sugawara H, Koike T, Lopez R, Gibson TJ, Higgins DG (2003). Multiple sequence alignment with the Clustal series of programs. Nucleic Acids Res.

[38] Tao J, Wang L, Liu D, Li Y, Bastin DA, Geng Y (2005). Molecular analysis of *Shigella boydii* O1 O-antigen gene cluster and its PCR typing. Can J Microbiol.

[39] Fratamico PM, Strobaugh TP (1998). Simultaneous detection of *Salmonella* spp and *Escherichia coli* O157:H7 by multiplex PCR. J Ind Microbiol Biot.

[40] Ågren J, Hamidjaja RA, Hansen T, Ruuls R, Thierry S, Vigre H (2013). In silico and *in vitro* evaluation of PCR-based assays for the detection of *Bacillus anthracis* chromosomal signature sequences. Virulence.

[41] Yu G, Stoltzfus A (2012). Population diversity of ORFan genes in *Escherichia coli*. Genome Biol Evol.

[42] Skippington E, Ragan MA: Phylogeny rather than ecology or lifestyle biases the construction of Escherichia coli-Shigella genetic exchange communities. Open Biol 2012, 2:120112. 10.1098/rsob.120112 PMC3472396 23091700

[43] Reid SD, Herbelin CJ, Bumbaugh AC, Selander RK, Whittam TS (2000). Parallel evolution of virulence in pathogenic *Escherichia coli*. Nature.

[44] Son I, Binet R, Maounounen-Laasri A, Lin A, Hammack TS, Kase JA (2014). Detection of five Shiga toxin-producing *Escherichia* coli genes with multiplex PCR. Food Microbiol.

[45] Feng P (1993). Identification of *Escherichia coli* serotype O157:H7 by DNA probe specific for an allele of uid A gene. Mol Cell Probes.

[46] Feng P (1997). Impact of molecular biology on the detection of foodborne pathogens. Mol Biotechnol.

[47] Feng P, Lampel KA (1994). Genetic analysis of uidA expression in enterohaemorrhagic *Escherichia coli* serotype O157:H7. Microbiology (Reading, Engl).

[48] Ochman HH, Lawrence JGJ, Groisman EAE (2000). Lateral gene transfer and the nature of bacterial innovation. Nature.

[49] Ito H, Kido N, Arakawa Y, Ohta M, Sugiyama T, Kato N (1991). Possible mechanisms underlying the slow lactose fermentation phenotype in Shigella spp. Appl Environ Microbiol.

[50] Pupo GM, Lan R, Reeves PR (2000). Multiple independent origins of Shigella clones of Escherichia coli and convergent evolution of many of their characteristics. P Natl Acad Sci USA.

[51] Martín MC, González-Hevia MA, Alvarez-Riesgo JA, Mendoza MC (2001). *Salmonella* serotype Virchow causing salmonellosis in a Spanish region. Characterization and survey of clones by DNA fingerprinting, phage typing and antimicrobial resistance. Eur J Epidemiol.

[52] Molina F, Jimenez-Sanchez A, Guzmán EC (1998). Determining the optimal thymidine concentration for growing Thy- *Escherichia coli* strains. J Bacteriol.

[53] Costas M, Sloss LL, Owen RJ, Gaston MA (1989). Evaluation of numerical analysis of SDS-PAGE of protein patterns for typing Enterobacter cloacae. Epidemiol Infect.

[54] Cheng LL, Li T-YT, Zhang YY (2004). Rapid preparation of total nucleic acids from *E. coli* for multi-purpose applications. J Biochem Mol Biol.

[55] Rozen SS, Skaletsky HH (2000). Primer3 on the WWW for general users and for biologist programmers. Methods Mol Biol.

[56] Untergasser A, Cutcutache I, Koressaar T, Ye J, Faircloth BC, Remm M (2012). Primer3 new capabilities and interfaces. Nucleic Acids Res.

[57] Altschul SFS, Madden TLT, Schäffer AAA, Zhang JJ, Zhang ZZ, Miller WW (1997). Gapped BLAST and PSI-BLAST: a new generation of protein database search programs. Nucleic Acids Res.

[58] Shen Z, Qu W, Wang W, Lu Y, Wu Y, Li Z (2010). MPprimer: a program for reliable multiplex PCR primer design. BMC Bioinformatics.

